# P02.73. Optimal healing environment in a continuing care retirement community: a feasibility study

**DOI:** 10.1186/1472-6882-12-S1-P129

**Published:** 2012-06-12

**Authors:** W Najm, R Chez, W Jonas, W Zhang

**Affiliations:** 1 Susan Samueli Center of Integrative Medicine-UC-Irvine, Newport Beach, USA; 2 Irvine, USA; 3 Samueli Institute, Alexandria, USA

## Purpose

The aim of this study is to evaluate if the overall quality of life of older adults living in an independent senior community can be positively impacted by weekly use of acupuncture directed at a specific complaint; the nightly use of lavender oil; and the daily use of guided imagery (GIM).

## Methods

This is an observational, before-after, 16-week study in which a convenience sample of 41 independent adults were recruited from a continuing care community retirement center “Regents Point”, located in Irvine, California. Subjects were competent adult volunteers, living at the facility. The study was divided into three periods: (1) Pre-intervention (4 weeks): baseline data were collected weekly prior to starting the intervention. (2) Active Intervention (8 weeks): data were collected weekly. Each subject received a vial of concentrated lavender oil and a prerecorded GIM MP3 player with 5 independent tracks [pain, stress, wellness, enhancing healing and recovery, and enhancing the immune system]. Acupuncture treatments were performed weekly targeting each person’s pain or highest health concern. (3) Post-intervention (4 weeks): data were collected weekly after the active intervention.

## Results

Twenty-six subjects (24 women; 2 men) completed the study. Age ranged from 74 to 95 years old. Statistical analysis comparing average results between the pre-intervention and post-intervention data identified a statistically significant difference in pain level (p<0.01), blood pressure (p=0.03); sleep quality (p=0.02), emotional well-being (p<0.02), and overall health (p=0.04). No significant changes were seen in physical health and in duration or onset of sleep.

## Conclusion

The positive results of this pilot study on blood pressure, pain, emotional well-being and overall general health are encouraging and suggest the need for a larger study to evaluate the impact of a multidisciplinary approach on general health and wellbeing.

